# Enhancing Plant Fibre-Reinforced Polymer Composites for Biomedical Applications Using Atmospheric Pressure Plasma Treatment

**DOI:** 10.3390/ma19030504

**Published:** 2026-01-27

**Authors:** Cho-Sin Nicole Chan, Wing-Yu Chan, Sun-Pui Ng, Chi-Wai Kan, Wang-Kin Chiu, Cheuk-Him Ng

**Affiliations:** 1 School of Professional Education and Executive Development, The Hong Kong Polytechnic University, Hong Kongjess.chan@cpce-polyu.edu.hk (W.-Y.C.); oscar.chiu@cpce-polyu.edu.hk (W.-K.C.);; 2 School of Fashion & Textiles, The Hong Kong Polytechnic University, Hong Kong; kan.chi.wai@polyu.edu.hk

**Keywords:** biomedical application, plasma treatment, jute fibre, epoxy, composite material, SEM, tensile properties, interlaminar shear strength

## Abstract

This research investigates the effects of corona plasma treatment on the mechanical properties of jute/epoxy-reinforced composites, particularly within biomedical application contexts. Plant Fibre Composites (PFCs) are attractive for medical devices and scaffolds due to their environmental friendliness, renewability, cost-effectiveness, low density, and high specific strength. However, their applications are often constrained by inferior mechanical performance arising from poor bonding between the plant fibre used as the reinforcement and the synthetic resin or polymer serving as the matrix. This study addresses the challenge of improving the weak interfacial bonding between plant fibre and synthetic resin in a 2/2 twill-weave-woven jute/epoxy composite material. The surface of the jute fibre is modified for better adhesion with the epoxy resin through plasma treatment, which exposes the jute fibre to controlled plasma energy and utilises dry air (plasma only), argon (Ar) (argon gas with plasma), and nitrogen (N_2_) (nitrogen gas with plasma) at two different distances (25 mm and 35 mm) between the plasma nozzle and the fibre surface. In this context, “equilibrium” refers to the optimal combination of plasma power, treatment distance, and gas environment that collectively determines the degree of fibre surface modification. The results indicate that all plasma treatments improve the interlaminar shear strength in comparison to untreated samples, with treatments at 35 mm using N_2_ gas showing a 35.4% increase in shear strength. Conversely, plasma treatment using dry air at 25 mm yields an 18.3% increase in tensile strength and a 35.7% increase in Young’s modulus. These findings highlight the importance of achieving an appropriate equilibrium among plasma intensity, treatment distance, and fibre–plasma interaction conditions to maximise the effectiveness of plasma treatment for jute/epoxy composites. This research advances sustainable innovation in biomedical materials, underscoring the potential for improved mechanical properties in environmentally friendly fibre-reinforced composites.

## 1. Introduction

Textile-based biomaterials are now widely used across routine medical practice, appearing in millions of clinical procedures and home care applications each year. Their extensive use in wound dressings, implantable meshes, vascular grafts, and wearable therapeutic devices highlights both their high clinical demand and their growing importance within the medical material sector [1,2,3]. Implantable vascular textiles—such as woven or knitted polyethylene terephthalate (PET) and expanded polytetrafluoroethylene (ePTFE)—account for hundreds of thousands of graft implantations annually, worldwide, and several million cumulatively over recent decades [4,5]. These examples reflect the broad and accelerating adoption of woven, knitted, braided, nonwoven, and electrospun textile architectures across a wide range of medical indications. Textile structures enable controlled porosity, mechanical compliance, and reinforcement, which directly influence their clinical performance. Woven or knitted PET (Dacron) and ePTFE vascular grafts utilise specific yarn geometries and fabric densities to balance suture retention, burst strength, and permeability [4,5]. Braided or knitted scaffolds made from ultra-high molecular weight polyethylene (UHMWPE), PET, or bioresorbable poly (L-lactic acid) (PLLA) are designed for ligament and tendon repair, where braiding angle and filament count modulate stiffness and fatigue life to mimic native fascicle behaviour [6]. Electrospun nanofibre dressings—such as polycaprolactone (PCL)/gelatin and chitosan-based systems—offer a large surface area for moisture regulation and provide a platform for delivering antimicrobials or bioactive compounds [7,8]. Lightweight, large-pore warp-knit polypropylene, or composite meshes used in hernia repair, are engineered to improve tissue integration and reduce the foreign body response [9,10]. Collectively, the combination of textile processing and fibre–matrix design enables the application-specific control of mechanical properties, structural architecture, and surface chemistry.

In recent decades, Polymer Matrix Composites (PMCs) reinforced by inorganic fibres, such as carbon, glass, or Kevlar, have been developed to offer numerous advantages, including superior specific stiffness and strength, impressive fatigue resistance, and excellent damping characteristics. These composites also demonstrate remarkable resistance against chemicals and outstanding electrical insulation capabilities [11]. Their vast applications span across various sectors, including but not limited to aviation, aerospace, industry and mining, transportation, and the construction sector. Despite their immense usefulness in various engineering applications, traditional inorganic fibre-reinforced polymer composites pose significant environmental challenges due to their inability to biodegrade. After their intended usage, these materials often find their way into landfills, causing substantial harm to the environment [12]. The rapid increase in plastic waste, coupled with the difficulty of recycling such materials, further compounds the environmental and resource-related issues that mankind currently faces. A potential solution to this issue lies in the development of green composites, which are made from natural, renewable, or biodegradable resources. Recent investigations have demonstrated the biodegradation potential of natural fibre-based composites. Wang et al. studied jute fibre-reinforced phylactic acid (PLA) composites and reported a progressive mass loss of about 20.2% over a three-month period, indicating measurable degradation within the matrix material [13]. In another study, Haque et al. observed that jute epoxy composites initially gained weight due to moisture uptake by the porous fibres; however, extended exposure ultimately resulted in weight loss as fibre deterioration occurred through swelling and microbial activity [14]. Collectively, these findings suggest that the integration of natural fibres, particularly when paired with biodegradable matrices, can lead to composite materials with improved environmental compatibility.

In light of growing environmental consciousness, Plant Fibre-Reinforced Polymer Composites (PFRPCs) have gained popularity in recent years [12]. Compared to conventional inorganic glass or carbon fibres, plant fibres such as sisal, jute, flax, hemp, kenaf, kapok, and more offer several advantages. The abundance of these renewable resources minimises the cost of raw material importation while also ensuring a reliable supply chain. These diverse plant fibres, when combined with a polymer resin as a matrix material, form composites that are exploited across various applications for their unique properties and versatility [15,16]. PFRPCs are favourable for their renewability, environmental compatibility, cost-effectiveness, lightness, and exceptional mechanical performance, demonstrating attributes such as recyclability, biodegradability, and superior specific strength [16]. These qualities have propelled their increased usage in sectors such as aerospace, structural engineering, automobiles, and biomedicine. However, the incomplete fusion and bonding of polymer resin in a PFRPC often result in weak mechanical properties in the composite material [17]. This is due to weak interfacial adhesion between the hydrophilic plant fibre and the hydrophobic synthetic polymer matrix, coupled with strong fibre-to-fibre interactions and strong intermolecular hydrogen bonding [18,19]. These factors contribute to poor resin absorption and weak surface structure in the reinforcement fibre, resulting in poor compatibility between the fibre and matrix materials. On the contrary, if strong adhesion across the interface can be achieved, the stresses applied to the composite material are effectively transferred from the matrix to the embedded fibre material. This adhesion facilitates better load-bearing capacity and strengthens the entire composite material. In addition, the strength of the interfacial adhesion has a causal relationship with the fracture mechanisms present in fibre-reinforced composite materials. To address this issue, chemical treatment of plant fibres has emerged as a strategy to improve the mechanical properties of the resulting polymer composites, considering the pivotal role of interfacial adhesive strength in the mechanical performance of the composites [20].

Chemical solutions such as alkali, silane, acetic acid, and benzoyl chloride are commonly used to eliminate hydroxyl (–OH) groups from plant fibres in order to reduce moisture sensitivity and enhance the interlocking between the fibre and matrix materials [21]. However, treating fibres with chemical solutions involves complex processing steps, including chemical extraction and recycling, which increases their overall cost. In response to growing sustainability concerns, research is shifting toward more eco-friendly and streamlined treatment techniques that minimise chemical use and reduce processing complexity. Plasma treatment represents such an environmentally benign approach, improving the mechanical properties of PFRPCs while using minimal chemicals and energy and generating no chemical waste. Plasma, the fourth state of matter, consists of ionised gas produced by high-frequency alternating current (AC) or direct current (DC) electrical sources. It generates ions (10–30 eV), electrons (0–10 eV), ultraviolet (UV) radiation (200 > λ > 400 nm), and vacuum UV radiation (λ < 200 nm) (3–40 eV) [20]. These physicochemical interactions modify material surfaces through several mechanisms: (1) surface cleaning, enhancing the effectiveness of coupling agents and adhesion; (2) material ablation, improving physical bonding; (3) surface cross-linking, strengthening the surface layer; and (4) free radical formation, altering the chemical structure. These modifications transform the structural and surface properties of lignocellulosic fibres, thereby improving their mechanical bonding with polymer matrices [22]. Plasma treatment may also expose the fibre surface to sequential streams of different treatment gases, such as oxygen [23], nitrogen [24], and helium [25], producing additional chemical modifications. These gas-specific interactions induce atom removal, bond scission, free radical formation, and cross-linking [22], further contributing to enhanced interfacial adhesion.

Plasma treatment has been consistently shown to enhance the mechanical properties of various PFRPCs. For instance, a study by Sever et al. found that radio-frequency (RF) plasma treatment increased the interlaminar shear strength of jute/high-density polyethylene (HDPE) composites by 189% [26]. Scanning electron microscopy (SEM) images revealed notable changes in surface morphology, including increased surface roughness, that likely contributed to this improved interlaminar strength [27]. Another study found that air–plasma treatment increased the tensile strength of wood fibre–polypropylene composites by 20% [28]. Kurniawan’s study reported a 45% increase in strength and an 18% increase in the stiffness of basalt/polylactic acid (PLA) composites after Atmospheric Pressure Glow Discharge (APGD) plasma treatment [29]. Atomic force microscopy (AFM) images showed significant morphological changes and increased surface roughness [29]. A recent study observed a 14% improvement in Young’s modulus and an 8.5% increase in the tensile strength of jute composite laminate following plasma treatment [30]. These findings collectively suggest that plasma treatment, in addition to improving mechanical properties, also induces beneficial morphological changes, further promoting the development of stronger and more durable fibre composites. Despite the demonstrated effectiveness of plasma-based surface modification in improving fibre–matrix adhesion, its implementation into composite manufacturing on an industrial scale has not been accomplished. The gap arises from its dependence on low-pressure plasma systems combined with chemically intensive treatments to achieve surface modification, which involve high capital costs, complex processing, and impose an environmental burden. Atmospheric pressure plasma (APP) treatment provides a viable approach to the solvent-free surface modification of fibres with minimal energy input. However, the influence of plasma gas chemistry under atmospheric conditions on the mechanical behaviour of woven natural fibres has not been studied. While previous studies have focused on the surface modification of commonly used plant fibres as a reinforcement in composites for improving adhesion and mechanical properties [18,19,20,21,22,23,24,25,26], the objective of this work is to investigate the effect of atmospheric pressure plasma (APP) treatment with different gases (air, argon (Ar), and nitrogen (N_2_)) on the mechanical behaviours (tensile strength, Young’s modulus, and interlaminar shear strength) of woven jute/epoxy composites. Jute fibre is chosen in the current study as jute is the second-most produced plant fibre, after cotton, worldwide [31].

## 2. Experiment

### 2.1. Materials

In this study, (i) jute fibres with a density of 1.46 g/cm^3^ and a linear yarn density of 250 tex were used as the reinforcing material, forming the base fibre structure for composite fabrication. These fibres were incorporated into (ii) a 2/2 twill-weave jute fabric with an areal weight of 290 g/m^2^ and yarn counts of 6 ends/cm in both the warp and weft directions (Easy Composites (Beijing) Ltd., Beijing, China). All jute fabrics were conditioned for at least 24 h at 20 ± 1 °C and 65 ± 2% relative humidity before atmospheric pressure plasma (APP) treatment and composite fabrication. For the polymer matrix, (iii) a low-viscosity epoxy system, IN2 Epoxy Infusion Resin (AT30 Slow) (Easy Composites EU B.V., Hengelo, The Netherlands), with a density of 1.065 g/cm^3^ and a viscosity of 325 mPa·s, was selected due to its suitability for vacuum-assisted resin infusion. To complete the matrix system, (iv) the corresponding IN2 amine hardener supplied by the manufacturer was used in the recommended mixing ratio to ensure the complete curing and consistent polymerisation of the jute/epoxy composite specimens.

### 2.2. Corona Plasma Treatment of Jute Fibres

**Figure 1:**
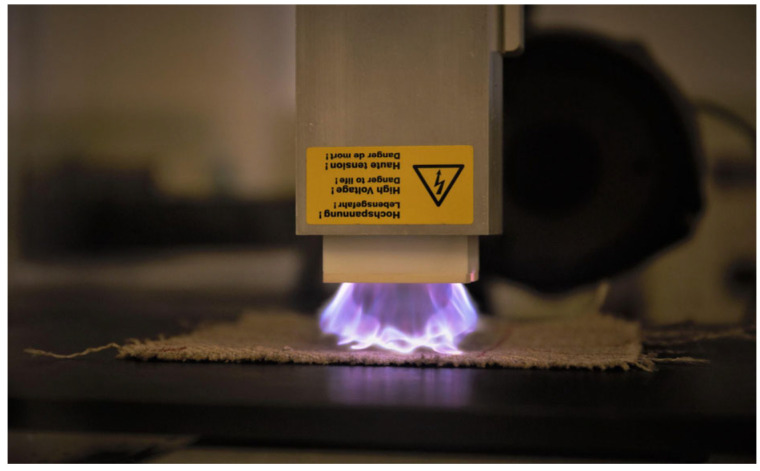
Plasma treatment process.

**Figure 2:**
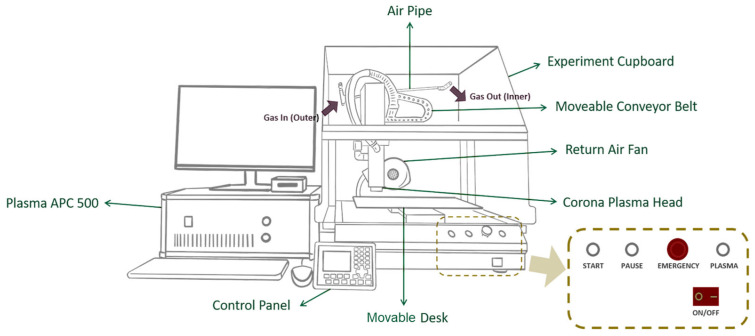
Plasma machine setup diagram.

The surfaces of jute fabrics were treated using the APP system (Corona Plasma System, model APC500; Diener Electronic GmbH + Co., KG, Ebhausen, Germany). This system employs a low-frequency generator operating at 40 kHz with a power output of 500 W to sustain the plasma within the reactor (Figure 1). The APP treatment was performed inside an enclosed, air-circulated chamber equipped with a movable conveyor belt and desk. Figure 2 shows the configuration of the APP system. The plasma nozzle head delivered a glow discharge onto the surfaces of the jute fabrics along the X and Y axes at a speed of 8.5 cm/s. Adjustable nozzle-to-fabric distances along the Z axis were set to 2.5 cm and 3.5 cm to allow a comparison of treatment outcomes. Three types of gases—dry air, argon (Ar), and nitrogen (N_2_)—were used separately as plasma sources during the APP treatments. Each gas was introduced into the chamber at a specified flow rate for 10 min, maintaining a pressure differential of 0.2–0.4 mbar. A summary of the sample identifiers and associated plasma treatment conditions is provided in Table 1.

### 2.3. Fabrication of Composite Materials

**Figure 3:**
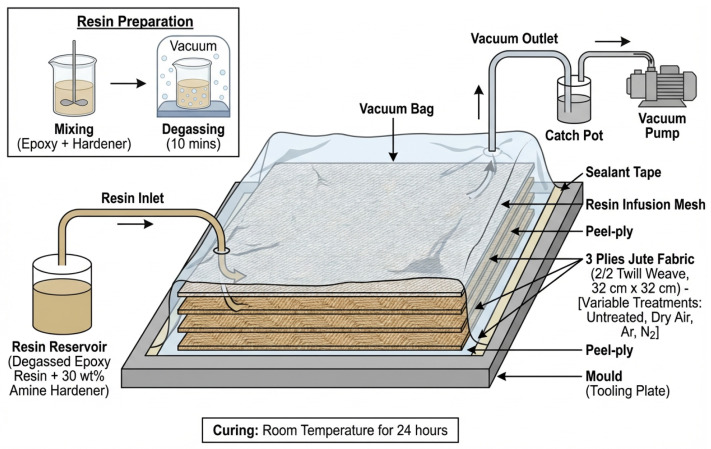
Jute/epoxy composite fabrication via vacuum-assisted resin infusion (VARI). The arrows indicate the direction of resin flow and vacuum during the infusion and curing process.

Jute/epoxy composite laminates were fabricated using the vacuum-assisted resin infusion method (Figure 3) [32]. Three plies of APP-treated 2/2 twill-weave-woven jute fabrics of 32 cm × 32 cm in size were covered by peel-ply layers on both sides and laid on a flat mould. Three types of APP-treated fabrics were used, including those treated with dry air, Ar, and N_2_. The peel-ply layers fully covered the jute fabrics in order to absorb excess epoxy resin during the infusion process. Resin infusion mesh was further applied to the top peel-ply layer to facilitate better resin flow. Vacuum bagging tape and vacuum bags were used to seal the mould completely. Control composite specimens were made with three plies of untreated jute fabrics, using the same fabrication procedures. Before infusing the epoxy resin, 30 wt% of amine hardener was mixed with the resin, and the mixture was degassed for 10 min in order to remove any trapped or dissolved gases. Following degassing, the mixed resin was infused under vacuum pressure to impregnate the fibre reinforcements. The setup was then allowed to be cured at room temperature for 24 h.

### 2.4. Characterisation Techniques

#### 2.4.1. Scanning Electron Microscopy (SEM)

The surface morphology of the jute fibre with and without APP treatment was examined using a Scanning Electron Microscope (SEM, HITACHI TM 4000 Plus, Hitachi, Tokyo, Japan). All fibre samples were uniformly gold-sputtered. This technique enabled the observation of different morphological features to identify the size and arrangement of individual fibres. The SEM operated by interpreting the electron beam diffraction patterns in proximity to the surface roughness of the fibres, and it also allowed the examination of the surface property modifications and chemical changes induced by plasma treatment, as plasma etching increases the active surface area of the treated material. The SEM analysis was performed at a voltage of 5 kV in secondary electron mode.

#### 2.4.2. Contact Angle Measurement

To examine the hydrophilic properties of jute fibres, contact angles were quantitatively measured using a Biolin Theta Contact Angle Meter (Biolin Scientific AB, Gothenburg, Sweden). A micro-syringe was used to precisely dispense 4 μL of distilled water onto the surface of each jute fabric sample. An image was captured for each experiment using a Charge-Coupled Device (CCD) camera built into the apparatus. To ensure representativeness, at least four measurements were taken from various locations on the fabric surface. The contact angles were calculated using the formula shown in Equation (1). The resulting values were subsequently plotted as a function of annealing time to assess the changes in surface properties.

(1) $$\;\gamma_{FA}=\gamma_{FL}+\gamma_{LG}cos\theta_{C}$$

where $\gamma_{FA}$ = surface tension at the fibre and air interface;

$\gamma_{FL}$ = interfacial surface tension between the fibre and liquid;

$\gamma_{LG}$ = surface tension between the fibre and liquid;

$\theta_{C}$ = contact angle (°).

#### 2.4.3. Tensile Test

Tensile tests were conducted using an MTS EXCEED Model E42 universal testing machine (Instron, Norwood, MA, USA) equipped with a 5 kN load cell. The tests were performed at a crosshead speed of 2 mm/min in accordance with ASTM D-3039 [33]. The mechanical properties reported in this study refer specifically to the jute-reinforced epoxy polymer composites, not the polymer matrix alone, ensuring a clear distinction between the composite system and its individual constituents. To ensure statistical reliability, six specimens of each type of APP-treated and untreated jute/epoxy composite were tested, and the resulting data were compiled and presented as average values. Using the stress–strain data, the average Young’s modulus and tensile strength were calculated to compare the mechanical performance of composites containing jute fibre reinforcement under different plasma treatment conditions.

#### 2.4.4. Interlaminar Shear Test

The interlaminar shear strength (ILSS) of the jute/epoxy composites was measured using a universal testing machine (HAIDA, model HD-B604-S, Dongguan, China) under a three-point bending load using the Short-Beam Shear Test method, in accordance with ASTM D2344/D2344M Standard [34]. Each type of specimen was cut into a short-beam shape with a size of 40 mm × 12 mm from the fabricated 32 cm × 32 cm composite laminates. Each specimen was placed on the three-point bend fixture, and a compressive load at a crosshead speed of 1.3 mm/min was applied to the middle of the specimen by a universal testing machine. The ILSS in this test represents the interfacial bonding strength between the jute fibre and epoxy resin in the composite specimens and was calculated using Equation (2), below. The average ILSS value for each type of APP-treated and untreated composite specimen was obtained from eight repeated measurements for comparison purposes.

(2) $$\;F^{sbs}=0.75\times \frac{P_{m}}{b\times h}$$

where $F^{sbs}$ = short beam strength (MPa);

$P_{m}$ = maximum load within the test process (N);

*b* = width of specimen (m);

*h* = length, respectively, of specimen (mm).

## 3. Results and Discussion

### 3.1. SEM Analysis

A comparative investigation of the morphology of the jute fibres before and after plasma treatment indicates substantial alterations. Plasma exposure induces the reorganisation of surface molecules, promoting their segmentation and interconnection. Ion bombardment reactions play a major role during this process. The ions generated within the plasma possess high momentum and a short mean free path, allowing them to interact effectively with the fibre surface. When these energetic ions collide with the jute fibres, they transfer energy that results in a distinct etching of the fibre surface and outer layers. This may lead to the formation of voids and micro-cracks as the exterior layers are stripped away or modified. The energetic interactions alter the molecular structure, breaking bonds and generating new surface characteristics that increase the availability of active bonding sites for matrix materials in composite systems. Consequently, ion bombardment reshapes the surface topography and enhances fibre functionality by improving matrix adhesion potential.

**Figure 4:**
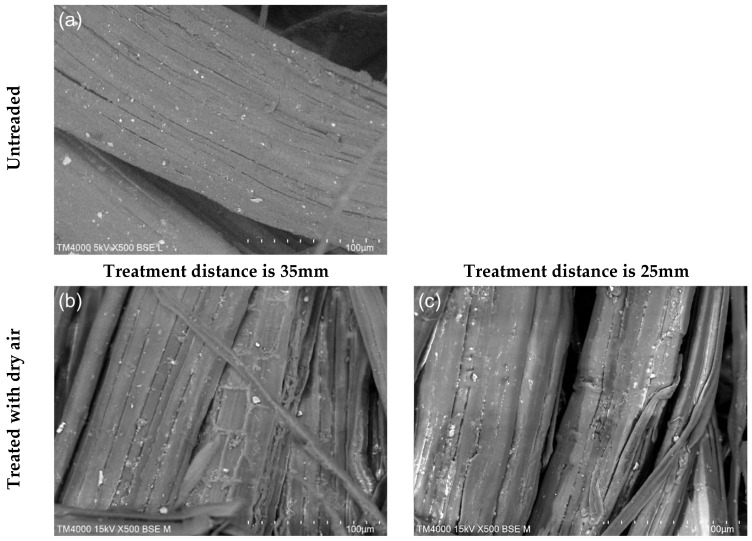

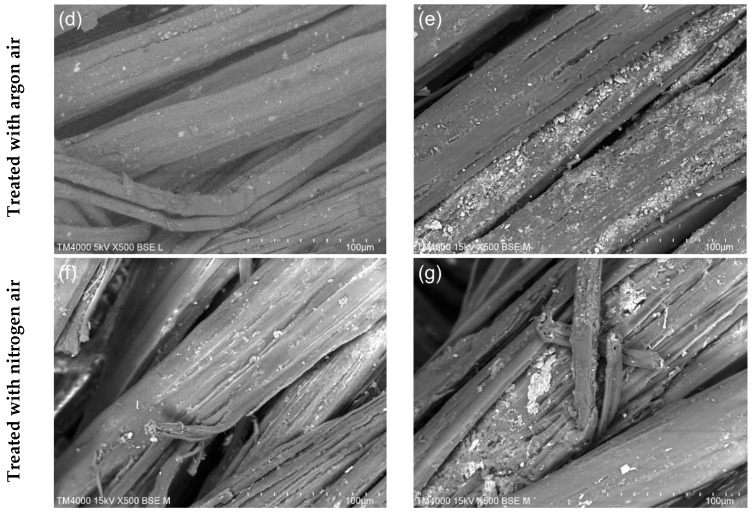
SEM micro-photographs of jute fabrics (500× magnification): (**a**) no treatment; (**b**–**g**) plasma treatment with dry air, Ar gas, N_2_ gas at plasma nozzle-to-fabric distances of 25 mm and 35 mm.

Figure 4 illustrates a comparison of jute fibres exposed to plasma treatment at consistent power levels with different nozzle heights and gas types. Untreated jute fibres retain a smoother and more uniform surface, whereas treated fibres display pronounced modifications. The treated fibres appear reduced in dimension and, in some cases, fractured, clearly indicating the purification of the fibre surface and the removal of non-cellulosic components or adhesive-like residues. This effect is particularly noticeable in samples treated with argon or nitrogen gases, where voids or scratch-like fragments are more prominent.

Increasing the distance between the plasma nozzle and the jute fibres from 25 mm to 35 mm results in more pronounced etching effects. The increased distance promotes wider plasma dispersion, yielding a more uniform and thorough surface alteration. This effect is most significant in argon-treated fibres, which show deeper micro-cracks and a higher density of surface discontinuities such as voids and fractured regions.

Argon plasma treatment is known for its strong etching effects, which generate deeper micro-cracks and a rougher fibre texture, increasing the surface area for mechanical interlocking. Although this improved roughness enhances interfacial adhesion, it may also reduce fibre strength due to the aggressive nature of the etching. Nitrogen plasma treatment, by contrast, tends to introduce functional groups that enhance chemical interactions while causing less physical damage, making it suitable where preservation of fibre strength is essential. Air plasma treatment introduces oxidative species that improve wettability and adhesion but generally causes milder surface texturing compared to argon and nitrogen. However, prolonged or excessive air plasma exposure can induce oxidative degradation, potentially weakening the fibres. All three treatments effectively enhance fibre–matrix adhesion, with the choice of gas determining the balance between improved bonding and potential loss of fibre integrity, as evidenced by the fractures and elongated surface cuts generated during etching. Therefore, atmospheric plasma treatment with argon, nitrogen, or air substantially modifies the jute fibre surface by etching and removing the smooth outer layers characteristic of natural plant fibres, which can otherwise hinder interactions with polymer matrices. The process increases surface activation and bonding capability, but excessive plasma intensity may compromise fibre strength even as adhesion improves.

### 3.2. Contact Angle (CA) Measurement

The measurement of the contact angle (CA) is an effective technique for assessing surface wettability prior to and during treatment activities. A reduced contact angle indicates increased hydrophilicity, implying enhanced wettability that promotes superior resin absorption during the infusion process. This feature can shift from hydrophilic to superhydrophilic, or from oleophilic to superoleophilic, depending on the surface roughness. Jute fibre, which often exhibits limited hydrophilicity due to surface contaminants, can achieve improved wettability and adhesion through plasma oxidation, which introduces reactive groups such as –OH, –OOH, and –COOH onto the fibre surface.

**Figure 5:**
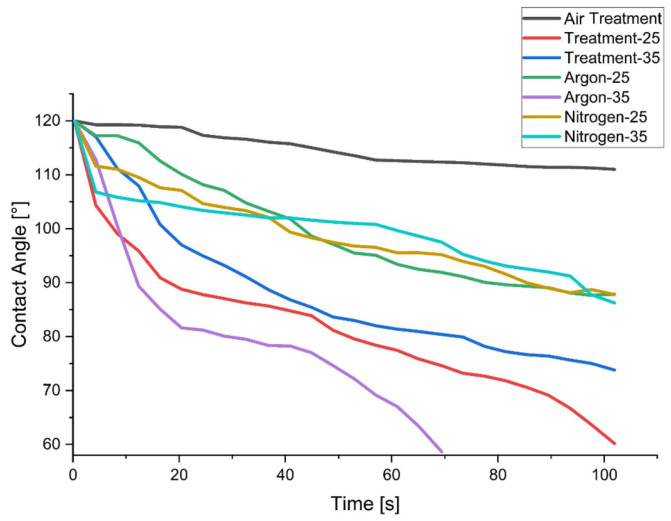
Contact angle test of treated and untreated jute fibre.

Figure 5 demonstrates the impact of the z-distance, defined as the distance between the plasma nozzle and the fibre surface, on the hydrophilicity of jute fibres. Measurements taken at 60 s show that argon gas treatment at a z-distance of 35 cm produced the greatest reduction in contact angle, achieving a 39.94% decrease and indicating a substantial enhancement in hydrophilic behaviour. Treatments conducted without gas at z-distances of 25 cm and 35 cm resulted in reductions of 30.61% and 27.08%, respectively. Argon treatment at 25 cm produced a 16.30% reduction, while nitrogen gas treatments at 25 cm and 35 cm yielded decreases of 14.39% and 10.70%, respectively. These results highlight the significance of z-distance in optimising plasma treatment to improve the surface characteristics of jute fibres, which is crucial for applications requiring enhanced moisture interaction and adhesion.

The mechanism driving these changes is the energetic breakdown of chemical bonds by plasma reactive species, which predominantly affects the amorphous regions of the fibre. These amorphous regions have a lower density compared to crystalline sections, making them more susceptible to modification. High-energy ions, electrons, and free radicals impact the fibre surface during treatment, providing sufficient energy to sever chemical bonds. This process removes less stable amorphous material while preserving the more stable crystalline structure, contributing to the changes observed in surface wettability [35].

### 3.3. Mechanical Properties

#### 3.3.1. Tensile Properties

**Figure 6:**
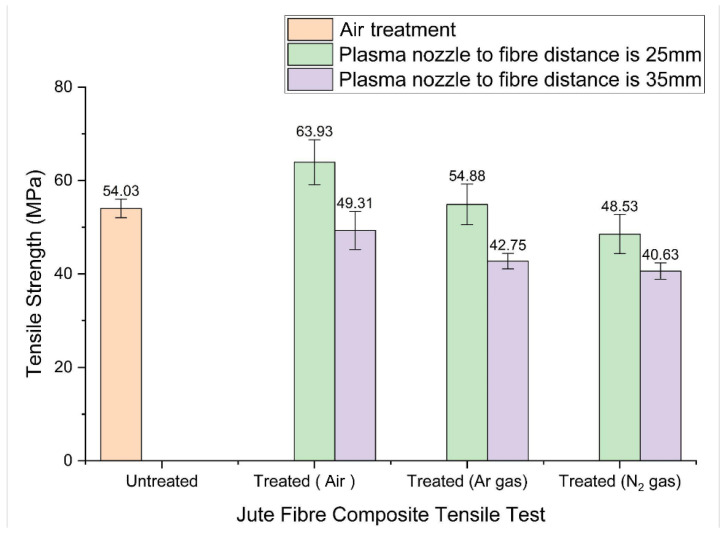
Tensile strength of treated and untreated jute composites.

The tensile properties of fibre-reinforced composites are critical indicators of performance and durability. A tensile test evaluates the resistance of the composite to deformation and fracture under tension. Plasma surface treatment can modify the physical and chemical characteristics of fibre surface layers, improving their reinforcement function within composites. However, excessive plasma exposure may adversely affect tensile performance. Because plasma treatment mechanisms vary according to treatment conditions, careful control is required to enhance composite performance while maintaining structural integrity. Figure 6 shows the tensile test results. The untreated composite exhibits a tensile strength of 54.03 MPa. When plasma treatment is applied at a reduced nozzle distance of 25 mm without supplementary gases, the tensile strength increases to 63.93 MPa, an improvement of 18.3%.

**Figure 7:**
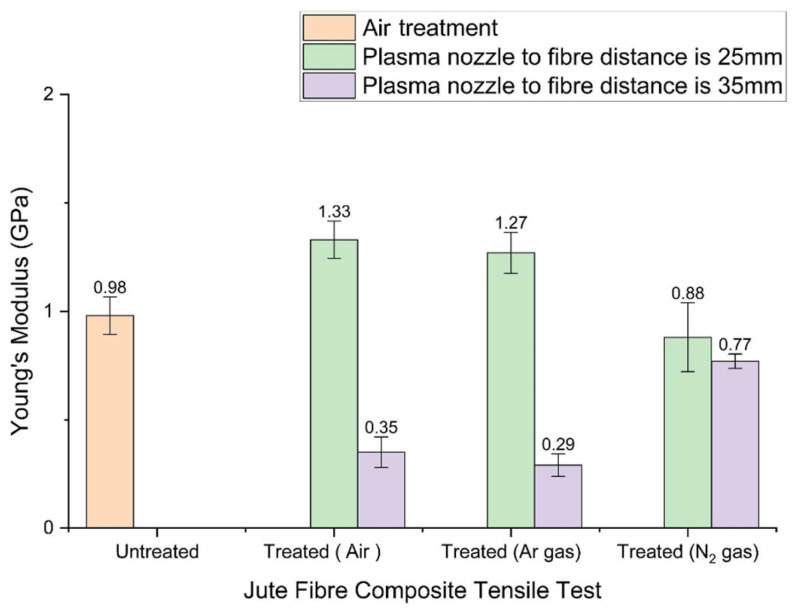
Young’s modulus in treated and untreated jute composites.

In contrast, when the nozzle distance is increased to 35 mm, the tensile strength decreases by 8.7%, 20.9%, and 24.8% for treatments without gas, with argon, and with nitrogen, respectively. These results indicate that treatment distance significantly influences tensile performance, and that gas-assisted treatments generally produce less favourable outcomes compared to treatments performed without added gases. Figure 7 presents the Young’s modulus values for both treated and untreated jute-reinforced composites. The Young’s modulus of the untreated composite is 0.98 GPa. When treated at a reduced distance of 25 mm, the modulus increases to 1.33 GPa (without gas) and 1.27 GPa (with argon), representing improvements of 35.7% and 29.6%. Conversely, treatments at 35 mm lead to reductions of 64.3%, 70.4%, and 21.4% for treatments without gas, with argon, and with nitrogen, respectively.

Overall, plasma treatments performed at a shorter distance (25 mm) and without gas provide the greatest improvements in both tensile strength and Young’s modulus. Gas-assisted treatments tend to produce more brittle failure modes due to increased surface damage. The type of plasma gas also influences mechanical outcomes. Treatments without supplementary gas rely on reactive oxygen- and nitrogen-containing species naturally present in air, which readily introduce functional groups that enhance fibre–matrix adhesion. Treatments using argon or nitrogen generate different reactive species; although effective in surface modification, these species can also create micro-cracks or excessive roughening, reducing the tensile strength and stiffness of the composite. In conclusion, both treatment distance and gas type markedly affect the mechanical properties of fibre-reinforced composites. Proper optimisation of these parameters is essential for achieving improved tensile performance suitable for structural applications

#### 3.3.2. Interlaminar Shear Strength (ILSS) Properties

**Figure 8:**
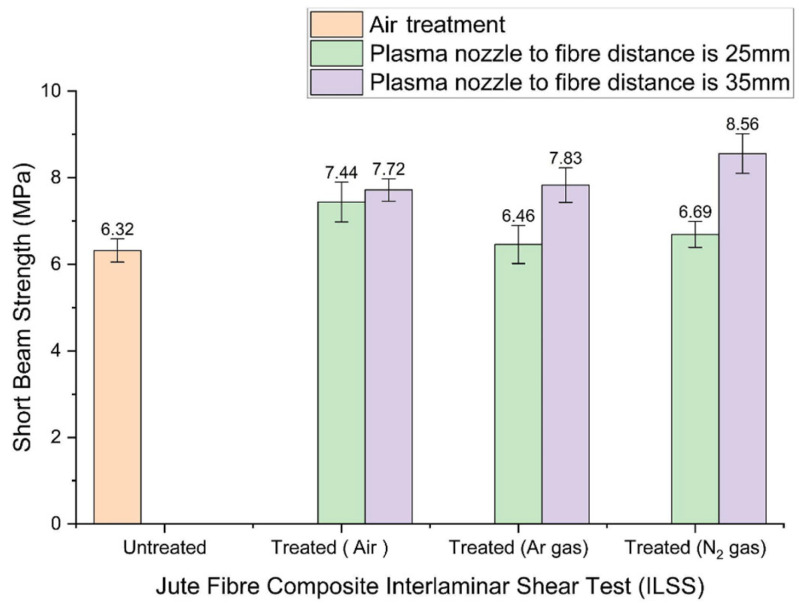
Short beam strength in treated and untreated jute composites.

Plasma surface treatment of fibres used in composite reinforcement enhances bonding strength between fibre and matrix, improving interfacial adhesion. A notable increase in interlaminar shear strength (ILSS) is therefore expected when fibre surfaces undergo effective plasma modification. Figure 8 shows the ILSS results. The untreated composite has an ILSS of 6.32 MPa. When the treatment nozzle is positioned at 35 mm, the ILSS increases significantly, particularly with nitrogen gas treatment, which reaches 8.56 MPa—a 35.4% improvement and the highest among all samples. Treatments at 35 mm using argon and without gas also show increases of 23.9% (7.72 MPa) and 22.2% (7.83 MPa), respectively. In contrast, treatments performed at 25 mm consistently show lower shear strengths.

This difference between tensile and shear responses arises from the distinct behaviours each test measures. Tensile strength depends primarily on fibre–matrix bonding and fibre integrity, both of which benefit from closer treatment distances. Shear strength, however, depends on the ability of the interface to resist sliding. Treatments at 35 mm—especially with nitrogen—appear to create surface features and chemical conditions that better resist shear stresses. Nitrogen treatment provides strong etching effects and favourable chemical activation, contributing to increased shear strength. Argon, despite producing notable surface roughening, may not remove cellulose and hemicellulose as effectively as nitrogen, resulting in less improvement in ILSS. Air plasma introduces reactive oxygen and nitrogen species that enhance wettability and can improve shear properties, though typically to a lesser extent than nitrogen.

The enhancement in interfacial adhesion observed after plasma treatment can be attributed to the removal or modification of surface layers rather than to changes verified through sugar analysis data. Fazeli M. demonstrated that only minor new FTIR features appeared following air plasma treatment, indicating that the enhancement in interfacial adhesion arises primarily from the removal or modification of cellulose- and hemicellulose-rich surface layers rather than from substantial chemical alterations [36]. Paiva et al. associated the C–H stretching region with cellulose and hemicellulose [37], while Rosa et al. reported that reduced band splitting in this region signifies the loss of lignin-like structures [38]. Consequently, plasma treatment exposes lignin-rich, more hydrophobic domains that interact more effectively with polymer matrices. Increased surface roughness generated by sputtering further enhances mechanical interlocking. Thus, the choice of plasma gas and treatment distance is critical in determining the ILSS performance of jute composites, highlighting the need for precise optimisation of the plasma treatment process.

## 4. Conclusions

The integration of innovative material technologies—such as plasma-treated jute fibre composites—can significantly advance biomedical applications where tailored mechanics, surface bioactivity, and durability are essential. In soft tissue fixation devices, orthopaedic screws/plates backings, resorbable splints, and porous scaffolds for tendon/ligament augmentation, improved fibre–matrix adhesion directly translates to better load transfer, reduced micro motion, and enhanced fatigue performance. The results of the study offer conclusive information about how plasma treatment affects the mechanical and surface qualities of jute fibres used in composite products.

The fibre surface is significantly altered by plasma treatment, which is typified by the breakdown of lignin present in these layers and the disappearance of smooth surface layers. This modification makes it easier for the fibre and matrix material to bind, which improves the composites’ tensile qualities considerably.

The type of gas used and the distance between the nozzle and the fibre during plasma treatment are important factors in the mechanical characteristics of the composites. Young’s modulus increases by 35.7%, and tensile strength increases by 18.3% when the distance is set at 25 mm, and no extra gas is employed. Tensile characteristics have been found to be adversely affected by treatments that involve increased nozzle lengths or the addition of nitrogen or argon gas.

Plasma treatment greatly improves the performance of fibre-reinforced composites in the area of interlaminar shear strength (ILSS). Shear strength is increased by 35.4% when nitrogen gas is added, and the treatment is carried out at a distance of 35 mm from the untreated composites. This data suggests that different properties are influenced differently by plasma treatment parameters, as it differs from the impacts seen on tensile properties.

These results provide useful recommendations for improving fibre-reinforced composites’ plasma treatment procedures. The importance of these parameters is highlighted by the notable impact that gas type and nozzle distance have on the mechanical characteristics of the composites. With improvements of 18.3% in tensile strength, 35.7% in Young’s modulus, and 35.4% in ILSS, these findings highlight how plasma treatment may enhance the functionality of composites reinforced with jute fibres.

## Figures and Tables

**Table 1:** Sample composition and plasma treatment settings.

Sample ID	Material Description	Plasma Source/Gas Environment	Distance (*Z*-Axis)	Treatment Duration
Untreated (Control)	2/2 twill-woven jute fabric	None	N/A	10 min
DA-25		Dry Air plasma	25 mm	
DA-35			35 mm	
Ar-25		Argon (Ar) plasma	25 mm	
Ar-35			35 mm	
N_2_-25		Nitrogen (N_2_) plasma	25 mm	
N_2_-35			35 mm	

## Data Availability

The original contributions presented in this study are included in the article. Further inquiries can be directed to the corresponding author.
